# Epistaxis in Rheumatism in Children

**Published:** 1902-03-01

**Authors:** 


### EPISTAXIS IN RHEUMATISM IN CHILDREN.

At a recent meeting of the Harveian Society Dr. Sidney Phillips drew attention to the frequency with which epistaxis is found in association with evidence of rheumatism in some form or another, an association which he regarded as too frequent to be put down to mere coincidence. After detailing a series of cases he said that the urine had been examined in all of them and was found to be free from albumin. There was no local nasal disease, and no cause could be found for the epistaxis except that there were evidences of a rheumatic diathesis. Many other cases could have been brought forward to illustrate the same point. In some of them the epistaxis came on with an attack of acute rheumatism, in others epistaxis alternated with attacks of articular inflammation, or was preceded by them, and in some cases it occurred in connection with chorea, which was presumably rheumatic. As is well known synovial effusion into joints and acute articular rheumatism may occur in children without any pain. Hence, with this clue in our minds, the occurrence of an attack of epistaxis may, on the one hand, suggest examination from other rheumatic symptoms, while, on the other hand, the discovery of such symptoms may be taken as explaining a perhaps puzzling occurrence of epistaxis in a child.

